# RELAY^**TM**^ Branched–International Results of Vessel Patency and Reintervention

**DOI:** 10.3389/fcvm.2022.962884

**Published:** 2022-06-29

**Authors:** Sidhant Singh, Abedalaziz O. Surkhi, Sven Z. C. P. Tan, Matti Jubouri, Damian M. Bailey, Ian Williams, Mohamad Bashir

**Affiliations:** ^1^Barts and The London School of Medicine and Dentistry, Queen Mary University of London, London, United Kingdom; ^2^Faculty of Medicine, Al-Quds University, Jerusalem, Palestine; ^3^Hull-York Medical School, University of York, Heslington, United Kingdom; ^4^Department of Vascular Surgery, University Hospital of Wales, Cardiff, United Kingdom; ^5^Neurovascular Research Laboratory, Faculty of Life Sciences and Education, University of South Wales, Treforest, United Kingdom; ^6^Vascular and Endovascular Surgery, Health Education and Improvement Wales, Wales, United Kingdom

**Keywords:** TEVAR, aortic arch, branched endograft, vessel patency, reintervention

## Abstract

**Background:**

Surgical intervention remains the mainstay treatment for aortic arch aneurysm and dissection, but the high mortality and morbidity rates have led to a need for the development of minimally invasive alternatives to arch reconstruction. RELAY™ Branched (Terumo Aortic, Inchinnan, UK) represents a viable option for complex endovascular aortic arch repair. We present multi-center data from Europe documenting the efficacy of the endograft in terms of its target vessel patency and reintervention rates.

**Methods:**

Prospective data collected between January 2019 and January 2022 associated with patients treated with RELAY™ single-, double-, and triple-branched endoprostheses from centers across Europe was retrospectively analyzed with descriptive and distributive analysis. Follow up data from 30 days and 6-, 12-, and 24 months postoperatively was included. Patient follow up was evaluated in terms of target vessel patency and reintervention rates.

**Results:**

Technical success was achieved in 147 (99.3%) cases. Over 24 months period, target vessel patency was maintained in 80.2% (*n* = 118) of patients. Target vessel cannulation was achieved in 146 (99.3%) cases. Over the 24-month follow-up period, 30 reintervention procedures were required, of which 29 (97%) took place within the South Europe region which accounted for 19.6% (*n* = 29) of total cases. Zero reinterventions were required in patients that were treated with single- or triple-branched endoprostheses.

**Discussion:**

The data presented herein demonstrates that RELAY™ Branched is a technically efficacious device for endovascular aortic arch repair and is associated with favorable target vessel patency and reintervention rates. Key design features of the endoprosthesis and good perioperative management can contribute greatly to mitigating reintervention and loss of vessel patency following endovascular aortic arch repair.

## Background

The pathologies of the thoracic aorta have been an area of great innovation in the last two decades focusing on reducing complications, interacting with complex anatomical situations and increasing ease of intervention. Since the introduction of the GORE TAG endograft for Thoracic Endovascular Aortic Repair (TEVAR) in 2005, endovascular intervention has become a promising avenue for treatment and has since become the preferred treatment for most thoracic aortic dissections and aneurysms (1). However, many centers and patients have continued to encounter relatively high complication rates and poor operative outcomes from the existing endografts due to issues with revascularization, stroke, graft patency and re-intervention rates (2). Although an open surgical approach remains the gold standard for total arch reconstruction (TAR), open surgery comes with its own set of risks and disadvantages. Patients must undergo cardiopulmonary bypass (CPB), and corresponding risks associated with general anesthesia and hypothermic circulatory arrest (HCA). Furthermore, the elderly and comorbid patients are at an even higher risk for surgical complications. In such cases endovascular repair is the safer and more promising option. The RELAY™ branched endoprosthesis allow for safer intervention and corresponding lower incidence of long-term complications (3).

Although there are many advantages for deployment of the RELAY™ branched device, there is need for clear clinical judgement when determining whether endovascular repair carries the best risk-benefit profile for patients. The criteria for RELAY™ branched graft placement in the aortic arch is chiefly based on the availability of a sufficient landing zone size and eligibility (or lack thereof) for open surgical repair.

Depending on the device used this varies slightly, but there should generally be at least 25 mm of viable aorta proximal to the dissection or aneurysm to safely plant the device (3). In cases where the vessel pathology is more proximal, patients could be considered for a Frozen Elephant Trunk (FET) operation, or custom made TEVAR endograft would suffice (3, 4).

The RELAY™ branched endoprosthesis offers significant advantages to patients of varying demographics. In addition to the benefits associated with minimally invasive intervention, patients with complicated anatomy which often result in compromised collateral circulation from single grafts can be safely revascularized with adequate entry tear coverage. With the options of single, double, and triple branched devices the RELAY™ device reduces invasiveness and risks by removing the need for aorto-cervical bypasses (4).

In the current study, we sought to assess the benefits of using the branched RELAY™ devices for aortic arch TEVAR while assessing the risk profile of the operation with a specific focus on vessel patency and re-intervention–two crucial metrics for assessing the appropriateness of endovascular repair. Vessel patency assesses the degree to which a vessel is not obstructed or leaking, which effectively summarizes the completeness of the vascular intervention. Re-intervention assesses the long-term efficacy of the intervention and whether patients need a secondary operation to treat the same issue again due to intervention failure (5).

## Device Design

The RELAY™ branched system is indicated for on-label use in patients with thoracic aortic aneurysms (TAAs) and penetrating atherosclerotic ulcers (PAUs), though use in patients with dissections and trauma to the aorta remains off-label (6). The device is designed specifically for the aortic arch, from zone 0 to zone 4, as a modular system deployed retrograde via access through the femoral artery or the iliac axis. The system includes a self-alignment mechanism, where the pre-curved introduction tip aligns itself with the curve of the aortic arch upon insertion (6, 7). This helps to decrease operative time and increases insertion accuracy contributing to overall improved outcomes (7). Windows designed for the supra-aortic branches are mounted to streamline accurate alignment with the arch branches and prevent occlusion of the left subclavian artery (LSA). Furthermore, the radio-opaque markers situated around the cannulation window clearly label an origin and where it aligns with the vessel branches, allowing for better orientation to the arch of the aorta improving intra-operative functionality of the branched stents (6). The large window size also allows for the addition of multiple branches accelerating cannulation without compromising cerebral perfusion (7). Moreover, the main tube of the device contains two internal connecting tunnels (posterior and anterior) which connect supra-aortic branches of the brachiocephalic trunk and the left common carotid artery extensions correspondingly (6). The system also contains a dual-sheath system which consists of a tougher outer sheath which aids delivery in tortuous iliac vessels and a flexible inner sheath to improve trackability and maneuverability even in acute and complicated cases. With the introduction of support wires and proximal collapsing, the device enables reduction of aortic instrumentation and achieves precise proximal landing (3, 6, 7).

## Methodology

### Study Design

Between January 2019 and January 2022, a retrospective European international multi-center investigation of TEVAR was conducted utilizing RELAY™. The information was collected prospectively and maintained in a database. Ethical review and approval was not required for this study with human participants, in accordance with the local legislation and institutional requirements.

### Patient Demographics

Between January 2019 and January 2022, a total of 148 TEVAR operations using RELAY™ were completed. This comprised 110 males and 38 females resulting in a M:F ratio of around 3:1. Patients with a mean age (IQR) of 70 (14.5) years were treated with RELAY™ for a variety of pathologies and differing levels of urgency (Table 1).

**Table 1:** Demographics.

**Gender (Male : female)**		3:1
**Mean age (IQR)**		70 (14.5)
**Pathology (%)**		
Aneurysm	107 (72.3%)	
Dissection	41 (27.7%)	
**Urgency (%)**		
Acute	68 (46%)	
Elective	80 (54%)	
**Total cases**		148

### Follow-Up Periods

All patients were followed up at 30 days, 6 months, 12 months, and 24 months postoperatively. Patients were evaluated at follow-up appointments for post-operative complications and disease progression. These factors included target vessel patency at the time of the appointment, and reinterventions conducted between the previous and current appointments. At the end of each follow-up period, overall mortality was recorded.

### Statistical Analysis

SPSS (IBM™ SPSS 28 for Windows) with the R plugin was used for all statistical analyses. A descriptive evaluation was carried out, and comparison investigations were performed where needed. In each analysis, propensity score matching was used to exclude any confounding variables. For normally distributed data confirmed by Shapiro Wilk W tests, the independent samples *t-*test was applied, and the the Mann-Whitney U served as the non-parametric equivalent. The Chi-Square method was used to determine differences in cumulative distribution frequency counts. Statistical significance for all two-tailed tests was set at *p* < 0.05.

## Results

### Operative Characteristics

The RELAY™ endoprosthesis was used to treat all patients and the average procedure time (IQR) was 258 (100) min. In one patient, technical success was not achieved, and the target vessel was not cannulated as a result. Another patient died after achieving technical success, and the target vessel could not be cannulated, though this mortality was not device-related. Table 2 summarizes operation parameters. Most patients' endovascular times were between 100 and 150 min. Table 3 shows the endovascular time groups. Tables 4–6 summarise the measured outcomes over the 24-month follow-up period.

**Table 2:** Operative characteristics.

Mean procedural time (IQR)		258 (100)
Branching number (%)		
	Single	17 (11.5%)
	Double	108 (73%)
	Triple	23 (15.5%)
Technical success (%)		147 (99.3%)
Target vessel cannulation (%)		146 (99.3%)^a^

a *Following percentages are calculated out of 147, one case died during the procedure, unrelated to the device*.

**Table 3:** Endovascular duration.

50–100	18
100–150	95
150–200	24
200–270	11

**Table 4:** Follow-up periods results.

	**30 Days**	**6 Months**	**12 Months**	**24 Months**
Vessel patency	147 (100%)	134 (91.1%)	124 (84.3%)	118 (80.2%)
Reinterventions	8 (5.4%)	6 (4.4%)	5 (4.0%)	5 (4.0%)
Deaths	4 (2.7%)	0 (0%)	0 (0%)	0 (0%)

**Table 5:** Relationship between branching number and vessel patency.

	**Single**	**Double**	**Triple**	**Total**	***p*** **-Value**
30 Days	16 (10.9%)	108 (73.4%)	23 (15.6%)	147 (100%)	-
6 Months	15 (10.2%)	96 (65.3%)	23 (15.6%)	134 (91.1%)	0.080
12 Months	16 (10.9%)	85 (57.8%)	23 (15.6%)	124 (84.3%)	<0.001
24 Months	15 (10.2%)	80 (54.4%)	23 (15.6%)	118 (80.2%)	0.001

**Table 6:** Relationship between branching number and reinterventions.

	**Single**	**Double**	**Triple**	**Total**	***p*** **-Value**
30 Days	0	8 (5.4%)	0	8 (5.4%)	0.005
6 Months	0	6 (4.4%)	0	6 (4.4%)	0.029
12 Months	0	5 (4.0 %)	0	5 (4.0 %)	0.020
24 Months	0	5 (4.0%)	0	5 (4.0%)	0.029

### Reintervention

All patients who eventually required reintervention during any of the follow-up periods were originally treated with a double branch stent. Significant differences in reintervention from the other types of branching was noted during the first 30 days, 6, 12, and 24 months after the procedure with *P* = 0.005, 0.029, 0.020, and 0.029, respectively.

During the first 30 days following the procedure, reintervention was required in 8 (5.4%) patients. Another 6 (4.4%) reinterventions were required during the following 6 months. During the subsequent 6 months (12-month follow-up), another 5 (4.0%) patients had undergone reintervention. All of them also required reintervention during the first 30 days and 8 of them during the second period. During the final follow-up period (24-months), another 5 (4.0%) reinterventions were recorded.

### Vessel Patency

A 100% (147) target vessel patency was recorded by the end of the first 30 days post-operatively. All patients treated with a triple stent (*n* = 23) exhibited lasting vessel patency throughout all follow-up periods. At 24 months follow up, over 80% of the cohort maintained target vessel patency.

At the 6-months follow-up appointment, a 91.1% (*n* = 134) of patients displayed target vessel patency. 93.7% (*n* = 16) of patients treated with single-branched device maintained vessel patency at 6 months, while 88% (*n* = 95) of those treated with a double branch stent had vessel patency at 6 months. A statistically significant difference in vessel patency in different branching groups was not seen during the 6 months follow-up period (*P* = 0.08).

At the 12-months follow-up appointment, 84.3% (*n* = 124) of the cohort exhibited target vessel patency. One hundred percent of the patients treated with a single-branched endoprosthesis displayed continued vessel patency at 12 months. Of the patients treated with a double branching stent 78.7% maintained target vessel patency. Target vessel patency at 12 months was different between branching number groups (*P* < 0.001).

By the 24-months follow-up period, 80.2% (*n* = 118) patients exhibited target vessel patency. This included 93.7% (*n* = 16) of the single-branched group, and 74% (*n* = 80) of the double-branched group.

## Discussion

Our multi-center data on endovascular repair of aortic arch pathologies (dissections and aneurysms) with the RELAY™ Branched System clearly demonstrates that the device is associated with excellent vessel patency and low re-intervention rates. In our cohort of 148 patients undergoing TEVAR with RELAY™ branched system for aortic aneurysm (*n* = 107) and aortic dissection (*n* = 41), technical success was achieved in 147 (99.3%) cases and target vessel patency was maintained in 147 (99.3%).

The RELAY™ Branched endoprosthesis has demonstrated success in establishing vessel patency across all of the devices, with a 30-day follow up demonstrating 100% (*n* = 147) vessel patency. These findings stand clear testament to the adaptability and suitability of the RELAY™ Branched device in adapting to the various anatomies across all cases. Over the 24-month review period, vessel patency fell to 91.1% (*n* = 134), 84.3% (*n* = 124) and 80.1% (*n* = 118) at 6, 12, and 24 months, respectively. It is likely the progression of the underlying vessel pathology was a contributing factor to the decrease in target vessel patency (5). The double branched group also experienced the greatest failure in target vessel patency falling by 25.9% from 73.4% (*n* = 108) to 54.4% (*n* = 80) over the 24-month period. Whereas, single branched only fell by 6.25% from 10.9% (*n* = 16) to 10.2% (*n* = 15) and tripled branched maintained 100% target vessel patency across the 24-month follow-up period.

The geographical distribution (see Tables 7–9) of cases across the European multi-center trial had significant impacts on the data collected. While Southern Europe comprised only 19.6% of the population (*n* = 29) it explained a disproportionate 95.8% (*n* = 23) of re-interventions required and experienced the greatest fall in vessel patency across the 24-month period, falling by 50% from *n* = 28 to *n* = 14. This could be linked to the observations of lower patency and higher re-intervention rates seen in double branched RELAY™ system deployments, as the Southern Europe sample population was made up of 96.6% (*n* = 28) patients receiving the double branched RELAY™ branched system. In view of this, it is reasonable to suggest that the loss of target vessel patency was not associated with RELAY™ Branched design; rather it is more likely associated with other exogenous factors including patient demographic and variations in center-to-center practice.

**Table 7:** Cases per region^a^.

West Europe	78 (52.7%)
East Europe	32 (21.6%)
South Europe	29 (19.6%)
North Europe	9 (6.1%)
Total	148

a *Based on UNSD Geoscheme*.

**Table 8:** Geographical distribution of reinterventions.

	**West**	**East**	**South**	**North**	**Total**	***p*** **-value**
30 Days	0	0	8	0	8 (5.4%)	<0.001
6 Months	1	0	5	0	6 (4.4%)	<0.001
12 Months	0	0	5	0	5 (4.0 %)	<0.001
24 Months	0	0	5	0	5 (4.0%)	<0.001

**Table 9:** Geographical distribution and vessel patency.

	**West**	**East**	**South**	**North**	**Total**	***p*** **-value**
30 Days	78	32	28	9	147 (100%)	-
6 Months	72	32	22	8	134 (91.1%)	0.017
12 Months	70	32	14	8	124 (84.3%)	<0.001
24 Months	65	31	14	8	118 (80.2%)	<0.001

Over the 24-month follow-up period a total of 24 re-interventions were required, however we must highlight that all these cases were of patients undergoing TEVAR with the double branched RELAY™ system. There were no re-interventions required for patients who received single and triple branched endoprosthesis. However, it is important to recognize that the patients receiving double branched RELAY™ systems constituted 73% (*n* = 108) of the population. Furthermore, the double branched group was also composed of the greatest percentage of acute patients 35.8% (*n* = 53). Collectively, these observations suggest that the RELAY™ branched system design and intra-operative aortic manipulation may not be the primary cause for reintervention; rather the complexity and progression of the vessel pathology are likely factors, either due to worsening vessel wall stability or increasing tortuosity and aortic pressure.

Re-interventions are a critical aspect of analysis when comparing TEVAR therapeutic options with the FET, the current gold standard for aortic arch reconstruction (8). As a new innovative treatment option for endovascular repair, aortic arch TEVAR provides many advantages over traditional open surgical intervention, not least the plethora of benefits associated with minimally invasive procedures (3). However, its advantages are being challenged with the current post operative reintervention rates observed (5). Zhang et al. observed an average reintervention rate of 14.6% post TEVAR in patients during their meta-analysis (5). They further identified the 3 main causes for post operative reintervention were type I endoleak, false lumen perfusion, and aortic dilation/new dissection. Type 1 endoleak is usually the result of insufficient proximal or distal landing seal zones in grafts, however, the RELAY™ branched endograft system offers the supra-aortic extensions via the internal connecting tunnels as an in-built mechanism to avoid the occurrence of endoleaks (3, 5). In cases where reintervention is required due to false lumen expansion, patients experience continuous perfusion of false lumen despite TEVAR; this could be due to graft failure or worsening vessel wall pathology regardless of stent placement (9).

Patients exhibiting postoperative disease progression are cause for greatest concern as they are at risk for late aortic related morbidity and mortality. Although distal re-entry tears can be sealed through an extension of the stent graft, there is a direct correlation between the length of aorta covered and the risk of spinal cord ischemia or even potential paralysis (10, 11). Decision making in these situations requires thorough planning and more evidence-based algorithm development to help ensure the correct therapeutic route is selected for patients. Risk factors for new dissection include patient age, prior interventions, and progression of vessel wall pathology (12). These factors can severely worsen a patient's condition and may result in the formation of a new tear and are very rarely associated with TEVAR or placement of a stent (5, 9).

Although branched systems like the RELAY™ branched endoprosthesis are designed to preserve and ensure target vessel patency after TEVAR, there are a range of complications that can occur to reduce post operative target patency in the mid-term. These include immediate occlusion of the target vessel due to coverage from the stent due to poor pre-operative planning, mal-alignment errors intra-operatively or incorrect graft sizing (13). Further late complications include migration of the graft, graft rotation and *in situ* thrombosis and stenosis arising due to intimal hyperplasia (2, 3).

In comparison to current market competitors the RELAY™ device demonstrates numerous advantages to both operator and patient. With the ability to withstand greater aortic contractile forces, without modification, endoprosthesis offers a single device solution. Competing devices like the Najuta™ endograft do not possess Z-stents between its first and second stent often required the use of a simple RELAY™ stent graft to provide adequate sealing from within, to ensure graft patency (14). Indeed, a recent systematic review found that a similar alternative–the Zenith Alpha endograft–carried a poorer technical success rate (96%) and a 13.3% (*n* = 92) reintervention rate, of which 26.1% (*n* = 24) patients required open surgical re-exploration (15). Additionally, the RELAY™ branched endoprosthesis provides a more effective delivery system. The dual sheath system and pre-curved introduction point allow for better alignment with the curvature of the aortic arch, thereby reducing manipulation difficulty and lowering surgical complications such as endoleaks (7). Toya et al. found that utilizing the RELAY™ branched endograft fits well and was adequate for landing in the compromised seal zone without introducing additional risks (16).

## Conclusion

The RELAY™ branched endoprosthesis has shown great promise as a new therapeutic adjunct to aortic arch TEVARs, as an innovative solution and proven alternative for patients that may not be suitable for open surgical repair. As an innovative procedure it has an anticipated steep learning curve, with the need for more evidence based algorithms to support patient selection criteria. The findings reported herein highlight the clinical efficacy and surgical safety of the RELAY™ branched endoprosthesis in treated aortic arch aneurysms and aortic dissection. This paper has emphasized the low re-intervention rates and great vessel patency obtainable through deployment of the device. It is an exceptional addition to the modern surgeon's armory in treating aortic arch vessel wall pathologies, complimenting existing gold standard treatments. The design and deployment technique of the device help promote faster and more precise arch repairs without compromising on desired surgical outcomes and reducing neurological complications. Further research and development of the device will help further reduce re-interventions and improve upon patency while flattening the learning curve through standardization of the device deployment technique.

## Data Availability Statement

The original contributions presented in the study are included in the article/supplementary material, further inquiries can be directed to the corresponding author.

## Ethics Statement

Ethical review and approval was not required for the study on human participants in accordance with the local legislation and institutional requirements. Written informed consent for participation was not required for this study in accordance with the national legislation and the institutional requirements.

## Author Contributions

SS, AS, and ST were responsible for drafting the manuscript. MJ, DB, IW, and MB were responsible for reviewing and providing feedback on the draft. All authors contributed to the article and approved the submitted version.

## Funding

DB was a Royal Society Wolfson Research Fellow (#WM170007).

## Conflict of Interest

On behalf of the South East Wales Vascular Network (DB and IW) and National Cardiovascular Research Network (DB). The remaining authors declare that the research was conducted in the absence of any commercial or financial relationships that could be construed as a potential conflict of interest.

## Publisher's Note

All claims expressed in this article are solely those of the authors and do not necessarily represent those of their affiliated organizations, or those of the publisher, the editors and the reviewers. Any product that may be evaluated in this article, or claim that may be made by its manufacturer, is not guaranteed or endorsed by the publisher.
